# Rare Case of Primary Anterior Abdominal Wall Abscess: Ultrasound, CT, and MRI Features

**DOI:** 10.7759/cureus.20618

**Published:** 2021-12-22

**Authors:** Parag S Mahajan, Jouhar J Kolleri, Hanan Farghaly

**Affiliations:** 1 Clinical Imaging Department, Hamad Medical Corporation, Doha, QAT; 2 Department of Laboratory Medicine and Pathology, Hamad Medical Corporation, Doha, QAT

**Keywords:** magnetic resonance imaging, computed tomography, ultrasound, abscess, anterior abdominal wall lesion

## Abstract

Primary anterior abdominal wall (AAW) abscess is a rare condition that can present clinically as an abdominal disorder and baffle even the most experienced clinicians. We discuss ultrasound, CT, and MRI features of a rare case of primary anterior abdominal wall abscess that was confirmed by histopathological findings.

## Introduction

Primary anterior abdominal wall (AAW) abscess is a rare pathology owing to the limited vascularity of the AAW. Most cases of AAW abscesses described in the literature are secondary to intra-abdominal pathology [1-6]. We could find only one case of primary actinomycosis of AAW [7], five cases of primary tubercular AAW abscesses [8-12], and one case of primary AAW abscess of unknown etiology [13] in the English literature. Primary AAW abscesses may appear as a solitary pathology without any intra-abdominal pathologic findings and may be associated with diabetes, immunocompromised status, etc. We present a case of a 78-year-old man who presented with abdominal mass which was further investigated and diagnosed to be a primary pyogenic AAW abscess which was incised and drained.

## Case presentation

A 78-year-old man presented to the emergency department of our hospital with generalized abdominal pain for about one month. It was associated with abdominal swelling, gradually increasing in size. The pain was aggravated by movement and not related to food intake. He had one spike of fever two weeks ago before the presentation and admission to the hospital. He also had a history of chronic cough. He was constipated and tolerating an oral diet. He was hypertensive, diabetic, with a history of coronary artery disease, on oral medication (including anti-coagulants), and compliant with the treatment. No history of nausea, vomiting, or urinary symptoms was noted.

On examination, his vital signs were as follows: temperature: 36.7 degrees Celsius, heart rate: 98/minute, respiratory rate: 18/minute, blood pressure: 116/58 mm Hg, oxygen saturation: 99%. On physical examination, he was conscious and oriented. There was a midline swelling on the abdomen, measuring around 15 x 10 cm with irregular margins. The swelling was fluctuant in nature, not fixed to the skin and adherent to abdominal muscles. There were skin colour changes with erythema and bruising. The swelling was minimally tender. No discharge from the swelling or any cutaneous scar was present. With contraction of the abdominal muscles, the swelling became fixed and more pronounced. There was no cough impulse (Fothergill’s sign). Basic laboratory tests were within normal limits. He was started on intravenous ampicillin/sulbactam, 1.5 g, four times daily for three days.

**Figure 1:**
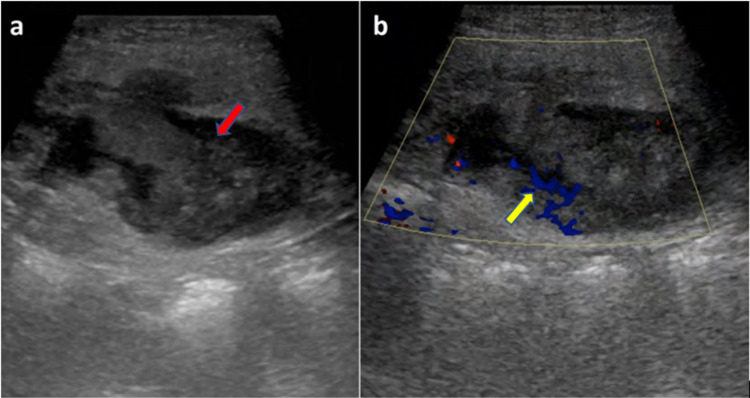
Ultrasound of abdomen showing a) a mixed solid and cystic lesion with irregular margins in the deep anterior abdominal wall in epigastrium with mild edema of adjoining soft tissue (Red arrow) and b) minimal peripheral vascularity (Yellow arrow).

Ultrasound of the abdomen was done which showed a mixed solid and cystic lesion with irregular margins in the deep anterior abdominal wall in the epigastrium in midline (Figure 1). Minimal peripheral vascularity was noted with no obvious internal vascularity. No movement of the lesion or its contents was observed. Edema of adjoining soft tissue of anterior abdominal wall was noted. Ultrasound features were suggestive of a complex cystic lesion. Differential diagnosis based on ultrasound were abscess and hematoma. Other rare differential diagnoses like mixed solid and cystic abdominal wall neoplasm, lymphovenous malformation, and cold abscess were also considered.

**Figure 2:**
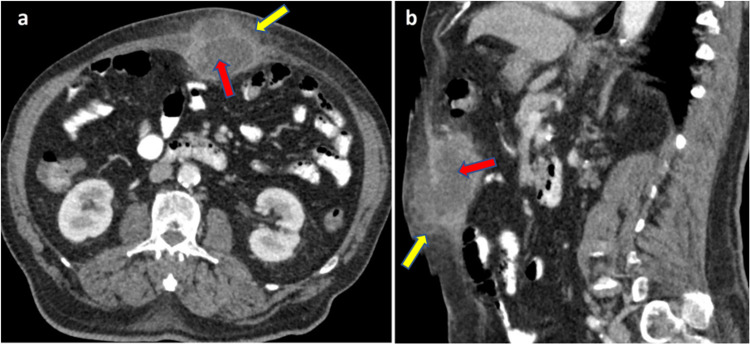
CT of abdomen with IV contrast, a) axial and b) sagittal reformatted image showing a large ill-defined complex hypodense lesion with irregular enhancing wall in supra-umbilical anterior abdominal wall in midline (red arrows) and associated surrounding fat stranding (yellow arrows).

CT scan of the abdomen with contrast demonstrated an ill-defined complex lesion with a large central hypodense component of fluid attenuation and irregular thick enhancing wall in the AAW in midline in the epigastric region located a few centimeters above umbilicus (Figure 2). It was primarily centered along linea alba with anterior extension into the deep subcutaneous plane and bulging posteriorly. Adjoining AAW showed diffuse fat stranding or edema. The findings were suggestive of abscess formation.

**Figure 3:**
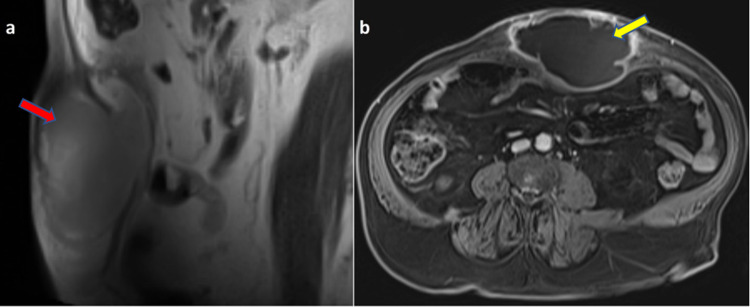
a) T2-weighted MRI sagittal plane showing a well-defined cystic lesion in the anterior abdominal wall (red arrow). b) T1-weighted post-contrast axial image showing midline cystic lesion in the anterior abdominal wall with irregular enhancing peripheral wall and few septations within (yellow arrow).

MRI scan of abdomen showed a large well-defined lesion in the AAW in the supraumbilical region in the midline, which is iso to hyperintense on T1 and T2-weighted images (Figure 3). On contrast study, irregular enhancing wall is noted along with few septations within. Diffusion-weighted images showed evidence of moderate restriction within this lesion. Thin rim of hypointensity was seen surrounding this lesion on T2-weighted and out-of-phase images. Mild fat stranding was seen in the adjacent soft tissue.

**Figure 4:**
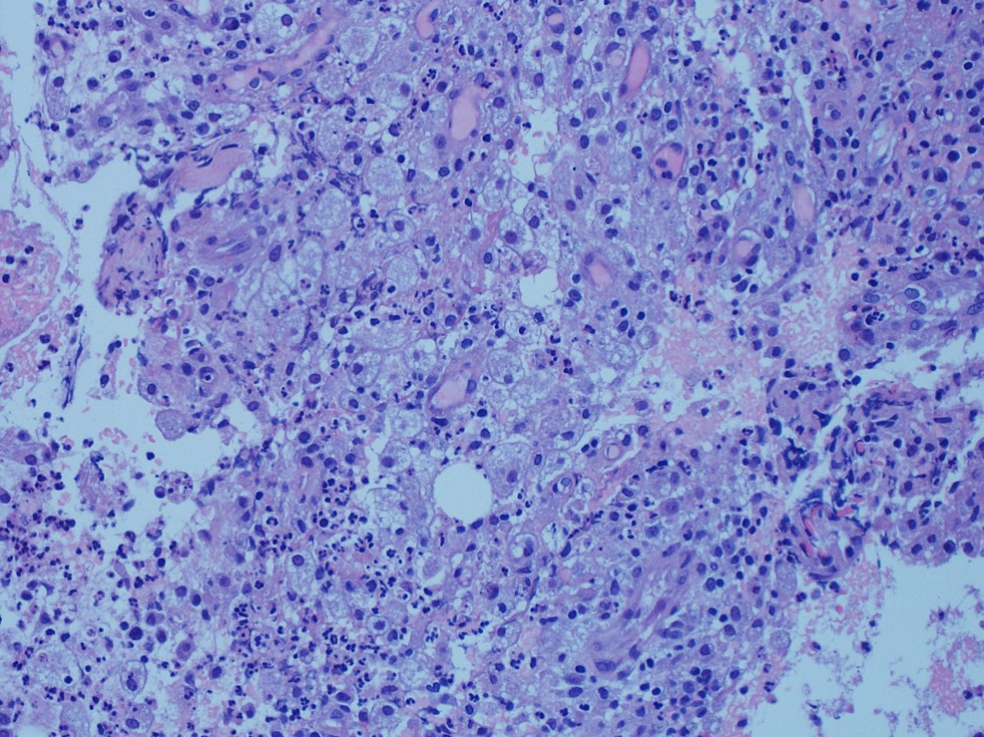
Histopathology image demonstrating fragments of subcutaneous tissue of abscess cavity containing acute inflammatory infiltrate with abundant neutrophils and necrotic debris. The abscess cavity was surrounded by chronic inflammatory infiltrate.

Wound culture result came as Streptococcus viridans, He was started on intravenous ceftriaxone, twice daily, for seven days. Ultrasound-guided aspiration of the midline AAW collection was done twice. Incision and drainage of the abscess cavity found a large pus cavity. Approximately 200 mL of foul-smelling greenish-colored pus was evacuated. Adjoining muscles were eroded and necrotic tissue was evident. Posterior rectus sheath was found to be intact. Histopathology demonstrated fragments of subcutaneous tissue of abscess cavity containing acute inflammatory infiltrate with abundant neutrophils and necrotic debris. The abscess cavity was surrounded by chronic inflammatory infiltrate (Figure 4). Histopathological diagnosis was pyogenic anterior abdominal wall abscess with inflamed subcutaneous tissue and granulation tissue with rare giant cell reaction. No evidence of granulomatous infection was found. Post-procedure hospital stay was uneventful, and he got discharged home with follow up in the surgical outpatient clinic.

## Discussion

Primary abscess of the AAW is very uncommon owing to its hypo vascularity. The diagnosis is established by exclusion of intra-abdominal organ involvement. Secondary anterior abdominal abscesses following colon carcinoma, acute pancreatitis, spilled gall stones, jejunal diverticulitis, tubercular liver abscess, Chron’s disease, etc. have been reported in the literature [1-6]. Only seven cases of primary AAW abscesses have been reported in the literature till date [7-13]. In our case, a pyogenic abscess developed in the anterior abdominal wall without any other infective pathology. Immunocompromised status, such as in cases of diabetes mellitus, steroid therapy, is a significant predisposing factor for primary AAW abscess.

Clinical features of AAW abscess include high spiking fever, chills, tachycardia, tachypnoea, leucocytosis, localised abdominal pain, etc. Weiner et al. reported a case of primary AAW abscess and made a literature review of secondary abdominal wall abscesses in 1975. According to the study, inflammatory process may not show the classic symptoms of inflammation clinically when the abscess is deep in the fascial planes of the abdominal wall, making the diagnosis more difficult. There may be little tenderness even if the mass is apparent and palpable. They also found that fever might be present or absent, and leucocytosis of varying levels is common [13].

Usually, AAW mass can be due to cellulitis (in diabetic patients) or hematoma (in anticoagulated patients after trivial trauma or chronic cough). Since our patient was on anticoagulants, there is a possibility that the patient developed an AAW hematoma following a bout of cough and due to his co-existing diabetes mellitus, it got infected from a systemic source.

The radiological examination of lesions of the abdominal wall helps to identify the nature of the disease. Palpation and other forms of physical examination frequently fail to specify the exact location and extent of a lesion. Forceful or repeated manipulation of abdominal wall masses should be avoided, if possible, as this might lead to rapid spread of the disease in case of malignant masses. The hallmark characteristics of an AAW abscess, such as mottled gas appearance and air fluid level, were earlier demonstrated using multiple X-ray views. X-ray was obtained tangentially to the lesion showing the soft tissue mass lateral to the preperitoneal fat line to determine if the lesion is most likely extraperitoneal. This exposed the patient to unnecessary radiation. Weiner et al. concluded that ultrasound B mode scanning was an accurate diagnostic test that was particularly useful in demonstrating disease of the anterior abdominal wall because it had no known adverse effects [13].

Abdominal wall imaging using ultrasound can be used to locate lesions that are clinically assumed to be within the abdominal cavity. Further investigations using CT and MRI help to assess the extent, depth of involvement, and to rule out other causes of abdominal abscess. These imaging modalities for diagnosing and draining intra-abdominal abscesses have resulted in a significant decrease in mortality. The most common cause of death is multiple organ failure. The severity of the underlying cause, a delayed diagnosis, poor drainage, and undetected foci of infection in the peritoneal cavity or elsewhere are all linked to death.

Panchagnula et al. presented a rare manifestation of colon cancer in a patient with AAW abscess. It demonstrated an abscess due to the perforation of a malignant mass, as well as bacteraemia and sepsis that followed. This article underlined the need for early detection and attribution of such an atypical finding to colorectal cancer as a differential diagnosis to prevent patient mortality due to sepsis in the hospital setting. If the abscess is accompanied by bowel symptoms, colonic carcinoma should be included in the differential diagnosis [1].

Treatment of primary AAW abscess consists of surgical incision and drainage under antibiotic cover and bed rest. Our patient was given intravenous ceftriaxone, twice daily for seven days and the abscess cavity was incised and drained. He got symptomatically better, hence discharged home and advised to follow up in surgical outpatient.

## Conclusions

With a thorough understanding of the pathophysiology of AAW abscesses and a high clinical index of suspicion, earlier detection, and apt treatment is possible which ultimately leads to lower morbidity and mortality. Diagnostic imaging like ultrasound, CT, and MRI allow non-invasive examination of the AAW, complementing the clinical evaluation of the patient.

## References

[1] Panchagnula K, Yalla P, Lakshminarayana B, Hegde K, Singaraddi R (2019). Anterior abdominal wall abscess: an unusual presentation of carcinoma of the colon. Arch Surg Clin Res.

[2] Kamble PM, Patil A, Jadhav S, Rao SA (2011). Anterior abdominal wall abscess with epididymo-orchitis: an unusual presentation of acute pancreatitis. J Postgrad Med.

[3] Eisenstat S (1993). Abdominal wall abscess due to spilled gallstones. Surg Laparosc Endosc.

[4] Sakurai Y, Tonomura S, Yoshida I (2005). Abdominal wall abscess associated with perforated jejunal diverticulitis: report of a case. Surg Today.

[5] Desai N, Patil S, Thakur BS, Das HS, Manjunath SM, Sawant P (2003). Abdominal wall abscess secondary to subcapsular tubercular liver abscess. Indian J Gastroenterol.

[6] Neufeld D, Keidar A, Gutman M, Zissin R (2006). Abdominal wall abscesses in patients with Crohn's disease: clinical outcome. J Gastrointest Surg.

[7] Hefny AF, Joshi S, Saadeldin YA, Fadlalla H, Abu-Zidan FM (2006). Primary anterior abdominal wall actinomycosis. Singapore Med J.

[8] Srivastava P, Gupta N, Mishra V, Misra SP, Dwivedi M (2021). Tuberculosis presenting as abdominal wall abscess in an immunocompetent adult. Int J Res Rev.

[9] Mitulkumar P, Shailesh P, Purvi D (2017). Cold abscess of the anterior abdominal wall: an unusual primary presentation. Eurorad.

[10] Malhotra MK (2012). Cold abscess of the anterior abdominal wall: an unusual primary presentation. Niger J Surg.

[11] Sahu SK, Rawat J, Sindhwani G, Raghuvanshi S (2008). Primary cold abscess of the anterior abdominal wall: an unusual site of presentation. Internet J Surg.

[12] Nuwal P, Dixit R (2007). Tuberculosis of rectus abdominis muscle. Indian J Chest Dis Allied Sci.

[13] Weiner CI, Diaconis JN (1975). Primary abdominal wall abscess diagnosed by ultrasound. Arch Surg.

